# Global carrier frequency and predicted genetic prevalence of patients with pathogenic sequence variants in autosomal recessive genetic neuromuscular diseases

**DOI:** 10.1038/s41598-024-54413-1

**Published:** 2024-02-15

**Authors:** Won-Jun Choi, Soo-Hyun Kim, Sung Rok Lee, Seung-Hun Oh, Seung Woo Kim, Ha Young Shin, Hyung Jun Park

**Affiliations:** 1 CHA University School of Medicine, Seongnam, Republic of Korea; 2 Department of Neurology, Gangnam Severance Hospital, Yonsei University College of Medicine, 211 Eonju-Ro, Gangnam-Gu, Seoul, 06273 Republic of Korea; 3 Department of Neurology, CHA Bundang Medical Center, CHA University, Seongnam, Republic of Korea; 4 Department of Neurology, Severance Hospital, Yonsei University College of Medicine, Seoul, Republic of Korea; 5 Rehabilitation Institute of Neuromuscular Disease, Gangnam Severance Hospital, Yonsei University College of Medicine, Seoul, Republic of Korea

**Keywords:** Genetic prevalence, Carrier frequency, Genome, Human, Neuromuscular disease, Pathogenic variant, Neurology, Neurological disorders, Clinical genetics

## Abstract

Genetic neuromuscular diseases are clinically and genetically heterogeneous genetic disorders that primarily affect the peripheral nerves, muscles, and neuromuscular junctions. This study aimed to identify pathogenic variants, calculate carrier frequency, and predict the genetic prevalence of autosomal recessive neuromuscular diseases (AR-NMDs). We selected 268 AR-NMD genes and analyzed their genetic variants sourced from the gnomAD database. After identifying the pathogenic variants using an algorithm, we calculated the carrier frequency and predicted the genetic prevalence of AR-NMDs. In total, 10,887 pathogenic variants were identified, including 3848 literature verified and 7039 manually verified variants. In the global population, the carrier frequency of AR-NMDs is 32.9%, with variations across subpopulations ranging from 22.4% in the Finnish population to 36.2% in the non-Finnish European population. The predicted genetic prevalence of AR-NMDs was estimated to be 24.3 cases per 100,000 individuals worldwide, with variations across subpopulations ranging from 26.5 to 41.4 cases per 100,000 individuals in the Latino/Admixed American and the Ashkenazi Jewish populations, respectively. The AR-NMD gene with the highest carrier frequency was *GAA* (1.3%) and the variant with the highest allele frequency was c.-32-13 T>G in *GAA* with 0.0033 in the global population. Our study revealed a higher-than-expected frequency of AR-NMD carriers, constituting approximately one-third of the global population, highlighting ethnic heterogeneity in genetic susceptibility.

## Introduction

Genetic neuromuscular diseases are clinically and genetically heterogeneous genetic disorders that primarily affect the peripheral nerves, muscles, and neuromuscular junctions. Clinical symptoms include muscle weakness, muscle atrophy, joint contractures, cardiomyopathy, exercise intolerance, sensory deficits, fatigue, myalgia, tremors, ataxia, and dysarthria. These symptoms exhibit heterogeneity according to the subtypes and causative genes. The application of high-throughput analysis has revolutionized the diagnosis of genetic neuromuscular diseases, enabling a more accessible and cost-effective approach^1^. To date, approximately 600 causative genes have been identified^2^. The incidence and prevalence of genetic neuromuscular diseases have been explored in many studies, and it has been observed that variations in methodologies and ethnicities contribute to substantial discrepancies in reported values^3–5^. To achieve a comprehensive understanding of the epidemiological landscape of genetic neuromuscular diseases on a global scale, a large-scale study using uniform analytic methods is urgently required.

Since the completion of the Human Genome Project^6^ several databases of human exomes and genomes have been created. Notably, the Genome Aggregation Database (gnomAD) is the most representable resource^7^. This database serves as a comprehensive repository for information from diverse exomes and genomes, offering valuable insights into human genetic variation. These data suggest that a certain percentage of individuals in the general population carry pathogenic or likely pathogenic variants (PLPVs) of a specific gene. It could provide crucial genomic data for predicting the carrier frequency and genomic prevalence of autosomal recessive Mendelian disorders including inherited retinal diseases, Pompe disease, congenital hypothyroidism, and Upshaw-Schulman syndrome^8–11^. This genetic information provides simple epidemiological information and plays a crucial role in various fields, including genetic counseling and drug development.

Therefore, our study aimed to identify PLPVs using genetic data sourced from the gnomAD database. Additionally, our objectives were to the calculate the carrier frequency and predict genetic prevalence of autosomal recessive neuromuscular diseases (AR-NMDs) across major ethnicities worldwide.

## Results

### Identification of PLPVs of AR-NMD genes

**Figure 1:**
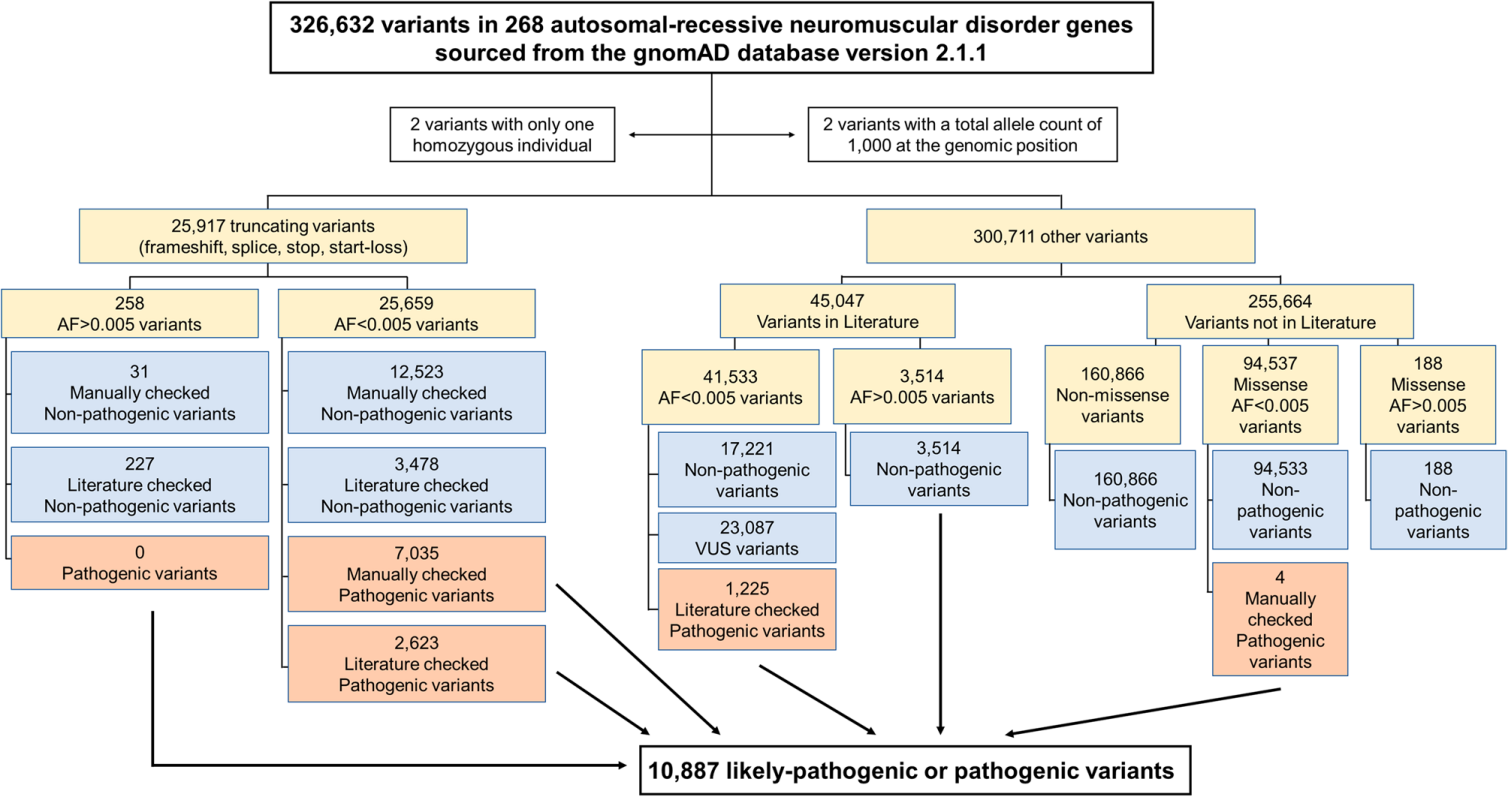
Flowchart depicting the analytical scheme for the variants sourced from the gnomAD database.

Figure 1 and Supplementary Table 2 illustrate the analytical scheme used to evaluate the AR-NMD gene variants. We identified a comprehensive set of 326,632 AR-NMD variants derived from the gnomAD database. We excluded two variants that were present in only one homozygous individual and were not found in heterozygotes, suggesting the potential unreliability of the reads. We also excluded two variants: c.5C>G in *SMN1* and c.359-1G>T in *TTPA*). Despite being considered PLPVs, the total allele counts at the genomic positions of the two variants were < 1000 (86 and 526, respectively). Subsequently, 326,628 variants were categorized into two main subgroups: 25,917 truncating variants and 300,711 non-truncating variants. Among the truncating variants, most (25,659) exhibited an allele frequency < 0.005. This analysis identified 9658 truncating PLPVs (2,623 literature verified variants and 7035 manually verified variants). Among the 300,711 non-truncating variants, 45,047 variants had references in scientific literature, whereas 255,664 lacked references. This analysis identified 1229 non-truncating PLPVs (1225 literature verified variants and 4 manually verified variants). In summary, a total of 10,887 PLPVs were identified, including 3,848 (35.3%) literature verified and 7039 (64.7%) manually verified variants (Supplementary Tables 2 and 3). Among the manually verified variants, 7035 were truncating and classified as PLPVs based on two pieces of evidence: (1) a null variant in a gene where the loss of function is a known mechanism of the disease, and (2) absent or an extremely low frequency in controls in the gnomAD database. The remaining four manually verified variants were missense variants and were classified as PLPVs based on three pieces of evidence: (1) the same amino acid change as a previously established pathogenic variant, (2) absent or extremely low frequency in controls in the gnomAD database, and (3) multiple lines of computational evidence supporting a deleterious effect on the gene or gene product. There were 22 homozygous PLPVs of AR-NMDs in the gnomAD database (Supplementary Table 4).

The allele frequency ratio of the literature verified PLPVs to total PLPVs is 60.4% in the global population. This allele frequency ratio was the highest in the ASJ (83.3%) population, followed by the NFE (65.0%), FIN (64.7%), AMR (59.4%), SAS (52.7%), EAS (51.4%), and AFR (50.3%) populations (Supplementary Table 5).

### Carrier frequency and predicted genetic prevalence of AR-NMDs

**Figure 2:**
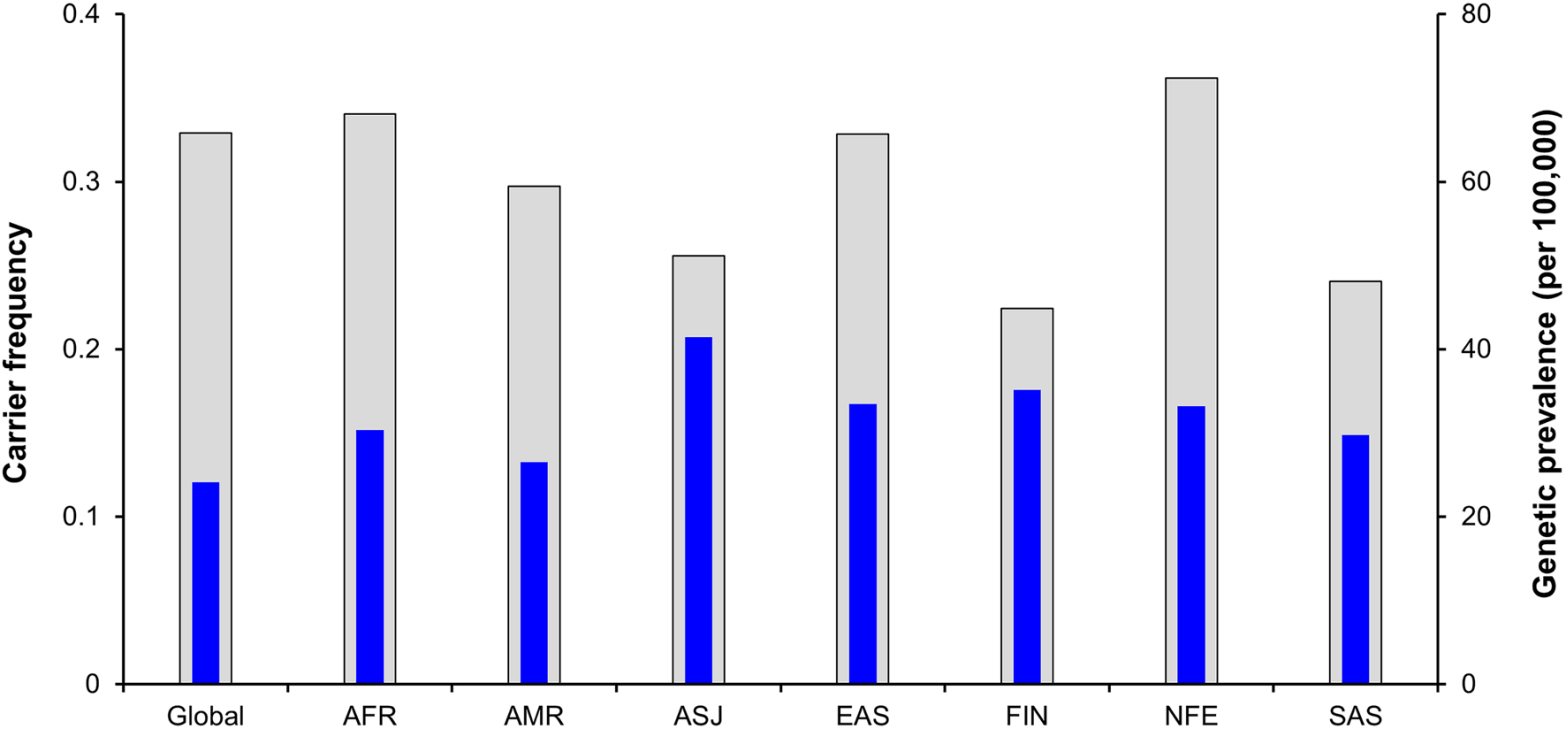
Global carrier frequency and predicted genetic prevalence of autosomal recessive neuromuscular diseases represented per subpopulation worldwide. The gray bars measured along the left vertical axis indicate the carrier frequency. The blue bars measured along the right vertical axis indicate the predicted genetic prevalence.

Figure 2 shows the carrier frequency and predicted genetic prevalence of AR-NMDs. The carriers of AR-NMDs are predicted to comprise 32.9% of the global population. Among subpopulations, the NFE population showed the highest carrier frequency at 36.2%, followed by AFR, EAS, AMR, ASJ, SAS, and FIN at 34.0%, 33.4%, 29.7%, 25.6%, 24.1%, and 22.4%, respectively. The predicted genetic prevalence of AR-NMD is estimated to be 24.3 cases per 100,000 individuals worldwide. The ASJ population had the highest predicted genetic prevalence of 41.4 cases per 100,000, followed by EAS, FIN, NFE, AFR, SAS, and AMR at 36.9, 35.2, 33.2, 30.3, 29.8, and 26.5 per 100,000 individuals, respectively.

### Common AR-NMD genes according to subpopulation

Table 1 and Supplementary Table 6 show the carrier frequency and predicted genetic prevalence for each AR-NMD gene in the different subpopulations. The *GAA* (1.3%) was the only gene with a carrier frequency exceeding 1% in the global population. However, the number of genes with a carrier frequency exceeding 1% varied among subpopulations. ASJ had the highest count, with four genes exceeding this threshold (*FKTN*, 1.7%; *GBE1*, 1.4%; *GAA*, 1.3%; and *PFKM*, 1.0%), followed by EAS with three (*GAA*, 1.6%; *SLC22A5*, 1.4%; and *NEB*, 1.1%), FIN with three (*GLE1*, 2.6%; *ANO5*, 1.4%; and *UBA5*, 1.2%), NFE with two (GAA, 1.7% and ANO5, 1.1%), AFR with two (*PGAM2*, 1.4% and *GAA*, 1.3%), AMR with one (*ANO5*, 1.4%), and SAS with one (*GNE*, 2.7%).

**Table 1:** Top 30 causative genes linked to autosomal recessive neuromuscular diseases.

Gene	Carrier frequency								Predicted genetic prevalence*							
	All	AFR	AMR	ASJ	EAS	FIN	NFE	SAS	All	AFR	AMR	ASJ	EAS	FIN	NFE	SAS
*GAA*	0.0131	0.0126	0.0085	0.0132	0.0158	0.0019	0.0170	0.0069	4.0798	3.9967	1.8087	4.3762	6.3238	0.0943	7.3339	1.2107
*ANO5*	0.0088	0.0029	0.0143	0.0029	0.0031	0.0143	0.0107	0.0038	1.9681	0.2151	5.2014	0.2104	0.2453	5.1315	2.8681	0.3590
*NEB*	0.0075	0.0081	0.0060	0.0033	0.0108	0.0072	0.0069	0.0056	1.4231	1.6577	0.8941	0.2715	2.9295	1.2879	1.1920	0.7907
*PYGM*	0.0059	0.0050	0.0065	0.0006	0.0038	0.0022	0.0078	0.0023	0.8720	0.6303	1.0715	0.0087	0.3712	0.1196	1.5296	0.1327
*LAMA2*	0.0055	0.0081	0.0080	0.0036	0.0054	0.0016	0.0055	0.0050	0.7690	1.6399	1.5977	0.3188	0.7362	0.0605	0.7636	0.6340
*CAPN3*	0.0054	0.0071	0.0048	0.0002	0.0081	0.0017	0.0061	0.0036	0.7423	1.2867	0.5733	0.0009	1.6341	0.0686	0.9489	0.3225
*GNE*	0.0049	0.0019	0.0007	0.0002	0.0041	0.0003	0.0027	0.0271	0.6071	0.0885	0.0140	0.0010	0.4235	0.0016	0.1862	18.3741
*ATM*	0.0049	0.0037	0.0039	0.0014	0.0049	0.0017	0.0061	0.0028	0.5981	0.3491	0.3732	0.0483	0.6091	0.0740	0.9342	0.1976
*ACADVL*	0.0047	0.0034	0.0018	0.0008	0.0036	0.0027	0.0071	0.0017	0.5508	0.2875	0.0823	0.0151	0.3250	0.1810	1.2782	0.0746
*DYSF*	0.0047	0.0070	0.0057	0.0010	0.0073	0.0007	0.0043	0.0037	0.5431	1.2204	0.8124	0.0235	1.3497	0.0115	0.4639	0.3402
*SPG11*	0.0046	0.0055	0.0026	0.0024	0.0058	0.0013	0.0053	0.0033	0.5253	0.7697	0.1715	0.1413	0.8378	0.0421	0.7131	0.2790
*SLC22A5*	0.0044	0.0028	0.0040	0.0012	0.0138	0.0015	0.0040	0.0037	0.4899	0.1916	0.3952	0.0346	4.8007	0.0585	0.3981	0.3508
*RAPSN*	0.0041	0.0009	0.0031	0.0041	0.0020	0.0010	0.0064	0.0023	0.4149	0.0198	0.2433	0.4120	0.0957	0.0230	1.0109	0.1282
*UBA5*	0.0040	0.0011	0.0018	0.0000	0.0006	0.0120	0.0051	0.0001	0.3910	0.0279	0.0825	0.0000	0.0093	3.6086	0.6612	0.0004
*FKRP*	0.0037	0.0010	0.0025	0.0010	0.0062	0.0037	0.0054	0.0006	0.3424	0.0274	0.1568	0.0245	0.9589	0.3459	0.7276	0.0086
*GBE1*	0.0036	0.0013	0.0031	0.0139	0.0008	0.0027	0.0039	0.0007	0.3251	0.0445	0.2345	4.8228	0.0141	0.1786	0.3905	0.0113
*GMPPB*	0.0036	0.0008	0.0021	0.0085	0.0019	0.0025	0.0046	0.0022	0.3197	0.0166	0.1144	1.8052	0.0936	0.1612	0.5357	0.1234
*GLE1*	0.0035	0.0010	0.0008	0.0000	0.0001	0.0255	0.0023	0.0014	0.3142	0.0231	0.0141	0.0000	0.0003	16.3270	0.1348	0.0471
*DOK7*	0.0034	0.0039	0.0024	0.0000	0.0022	0.0009	0.0046	0.0020	0.2890	0.3829	0.1460	0.0000	0.1248	0.0209	0.5226	0.1031
*MYO18B*	0.0032	0.0037	0.0061	0.0002	0.0035	0.0008	0.0032	0.0027	0.2595	0.3462	0.9284	0.0010	0.3043	0.0147	0.2566	0.1772
*VWA3B*	0.0031	0.0050	0.0019	0.0004	0.0043	0.0003	0.0026	0.0044	0.2413	0.6229	0.0892	0.0038	0.4556	0.0020	0.1680	0.4929
*SACS*	0.0030	0.0040	0.0017	0.0014	0.0020	0.0013	0.0033	0.0022	0.2320	0.4114	0.0753	0.0509	0.0961	0.0434	0.2780	0.1181
*AGL*	0.0030	0.0045	0.0045	0.0008	0.0019	0.0006	0.0037	0.0018	0.2248	0.5024	0.5157	0.0159	0.0904	0.0081	0.3469	0.0849
*ADSS1*	0.0028	0.0009	0.0032	0.0020	0.0028	0.0013	0.0038	0.0013	0.2007	0.0182	0.2629	0.0989	0.1909	0.0398	0.3524	0.0401
*PNKP*	0.0028	0.0018	0.0036	0.0000	0.0032	0.0009	0.0033	0.0022	0.2006	0.0807	0.3260	0.0000	0.2609	0.0208	0.2744	0.1257
*AP5Z1*	0.0028	0.0031	0.0022	0.0088	0.0028	0.0009	0.0026	0.0016	0.1961	0.2441	0.1224	1.9299	0.1943	0.0215	0.1648	0.0610
*HEXB*	0.0028	0.0012	0.0055	0.0000	0.0021	0.0004	0.0036	0.0019	0.1915	0.0366	0.7703	0.0000	0.1126	0.0046	0.3324	0.0897
*POMGNT1*	0.0027	0.0011	0.0019	0.0004	0.0018	0.0051	0.0033	0.0014	0.1866	0.0281	0.0913	0.0039	0.0832	0.6400	0.2717	0.0479
*COQ8A*	0.0026	0.0016	0.0014	0.0056	0.0022	0.0037	0.0029	0.0009	0.1697	0.0604	0.0488	0.7704	0.1210	0.3353	0.2039	0.0219
*CHRNG*	0.0025	0.0027	0.0017	0.0004	0.0021	0.0021	0.0028	0.0025	0.1584	0.1881	0.0727	0.0039	0.1137	0.1152	0.2025	0.1623

*AFR* African/African American, *AMR* Latino/Admixed American, *ASJ* Ashkenazi Jewish, *EAS* East Asian, *FIN* Finnish European, *NFE* non-Finnish European, *SAS* South Asian.

*Predicted genetic prevalence per 100,000 individuals.

### Allele frequencies of PLPVs of AR-NMD genes

Table 2 and Supplementary Table 3 show the allele frequency of each PLPV associated with AR-NMDs in the major subpopulations. The variant with the highest allele frequency was c.-32-13T>G in *GAA* with 0.0033 in the global population. Within the subpopulations, the PLPV with the highest allele frequency was c.2179G>A in *GNE*, reaching 0.0133 in SAS, followed by c.433-10A>G in *GLE1* at 0.0118 in FIN; c.1167dupA in *FKTN* at 0.0078 in ASJ; c.233G>A in *PGAM2* at 0.0068 in AFR; c.-32-13T>G in *GAA* at 0.0053 in NFE; c.1385-42G>C in *AGRN* at 0.0028 in EAS; and c.-32-13T>G in *GAA* at 0.0027 in AMR.

**Table 2:** Allele frequency of top 30 pathogenic or likely pathogenic variants linked to autosomal recessive neuromuscular disease genes.

Genes	Reference transcript	Transcript consequence	Protein consequence	Verification	Allele frequency							
					All	AFR	AMR	ASJ	EAS	FIN	NFE	SAS
*GAA*	NM_000152	c.-32-13T>G	–	Literature	0.0034	0.0009	0.0027	0.0055	0.0002	0.0002	0.0053	0.0019
*UBA5*	NM_024818	c.1111G>A	p.A371T	Literature	0.0019	0.0005	0.0007	0.0000	0.0000	0.0060	0.0025	0.0000
*RAPSN*	NM_005055	c.264C>A	p.N88K	Literature	0.0015	0.0002	0.0009	0.0020	0.0000	0.0004	0.0026	0.0008
*PYGM*	NM_005609	c.148C>T	p.R50*	Literature	0.0015	0.0007	0.0014	0.0001	0.0001	0.0005	0.0025	0.0000
*GNE*	NM_00128227	c.2179G>A	p.V727M	Literature	0.0015	0.0000	0.0000	0.0000	0.0000	0.0000	0.0000	0.0133
*ACADVL*	NM_001270448	c.848T>C	p.V283A	Literature	0.0012	0.0001	0.0002	0.0002	0.0000	0.0011	0.0022	0.0001
*FKRP*	NM_024301	c.826C>A	p.L276I	Literature	0.0011	0.0003	0.0003	0.0000	0.0000	0.0011	0.0023	0.0000
*ANO5*	NM_213599	c.191dup	p.N64Kfs*15	Literature	0.0010	0.0005	0.0004	0.0000	0.0000	0.0000	0.0020	0.0000
*GLE1*	NM_001003722	c.433-10A>G	–	Literature	0.0010	0.0000	0.0000	0.0000	0.0000	0.0118	0.0001	0.0000
*ANO5*	NM_213599	c.692G>T	p.G231V	Literature	0.0010	0.0002	0.0026	0.0003	0.0000	0.0001	0.0012	0.0000
*GBE1*	NM_000158	c.691 + 2T>C	–	Literature	0.0009	0.0000	0.0001	0.0002	0.0000	0.0010	0.0014	0.0000
*DOK7*	NM_173660	c.1124_1127dup	p.A378Sfs*30	Literature	0.0007	0.0002	0.0008	0.0000	0.0000	0.0001	0.0012	0.0003
*ANO5*	NM_213599	c.2272C>T	p.R758C	Literature	0.0007	0.0000	0.0001	0.0000	0.0000	0.0055	0.0003	0.0000
*GMPPB*	NM_021971	c.79G>C	p.D27H	Literature	0.0007	0.0001	0.0000	0.0000	0.0000	0.0010	0.0012	0.0000
*PGAM2*	NM_000290	c.233G>>A	p.W78*	Literature	0.0006	0.0068	0.0002	0.0000	0.0000	0.0000	0.0000	0.0000
*POMGNT1*	NM_001243766	c.1539 + 1G>A	–	Literature	0.0006	0.0001	0.0001	0.0001	0.0000	0.0020	0.0009	0.0002
*ADSS1*	NM_152328	c.36del	p.G13Afs*21	Manual	0.0006	0.0000	0.0000	0.0000	0.0000	0.0005	0.0012	0.0000
*HEXB*	NM_000521	c.1250C>T	p.P417L	Literature	0.0006	0.0000	0.0001	0.0000	0.0010	0.0001	0.0010	0.0001
*VWA1*	NM_022834	c.62_71dup	p.G25Rfs*74	Literature	0.0005	0.0007	0.0000	0.0041	0.0000	0.0000	0.0006	–
*CYP7B1*	NM_004820	c.1456C>T	p.R486C	Literature	0.0005	0.0002	0.0000	0.0001	0.0001	0.0004	0.0010	0.0000
*ANO10*	NM_018075	c.132dup	p.D45Rfs*9	Literature	0.0005	0.0001	0.0000	0.0000	0.0000	0.0006	0.0008	–
*MYO9A*	NM_006901	c.5093A>G	p.D1698G	Literature	0.0005	0.0000	0.0003	0.0000	0.0000	0.0000	0.0004	0.0022
*CLTCL1*	NM_007098	c.988G>A	p.E330K	Literature	0.0005	0.0002	0.0009	0.0000	0.0000	0.0000	0.0007	0.0001
*SGCA*	NM_000023	c.229C>T	p.R77C	Literature	0.0005	0.0001	0.0001	0.0000	0.0001	0.0019	0.0005	0.0000
*SORD*	NM_003104	c.458C>A	p.A153D	Literature	0.0005	0.0002	0.0001	0.0030	0.0000	0.0000	0.0006	0.0000
*SLC22A5*	NM_003060	c.136C>T	p.P46S	Literature	0.0004	0.0000	0.0001	0.0006	0.0000	0.0001	0.0008	0.0000
*ALS2*	NM_020919	c.3182 + 2T>G	–	Manual	0.0004	0.0007	0.0002	0.0002	0.0002	0.0011	0.0005	0.0000
*SORD*	NM_003104	c.757del	p.A253Qfs*27	Literature	0.0004	0.0006	0.0003	0.0000	0.0002	0.0005	0.0005	0.0001
*EXOSC3*	NM_016042	c.395A>C	p.D132A	Literature	0.0004	0.0003	0.0002	0.0000	0.0000	0.0000	0.0007	0.0001
*TPP1*	NM_000391	c.509-1G>C	–	Literature	0.0004	0.0000	0.0000	0.0000	0.0000	0.0002	0.0008	0.0000

*AFR* African/African American, *AMR* Latino/Admixed American, *ASJ* Ashkenazi Jewish, *EAS* East Asian, *FIN* Finnish European, *NFE* non-Finnish European, *SAS* South Asian.

### gnomAD individuals with homozygous PLPVs in AR-NMD genes

To determine whether gnomAD included individuals with AR-NMDs, we compared the number of gnomAD individuals with homozygous PLPVs and the expected number of individuals with homozygous PLPVs calculated using the predicted genomic prevalence. We identified 28 gnomAD individuals with homozygous PLPVs in the AR-NMD genes (Supplementary Table 4). However, the expected number of individuals with homozygous variants using the predicted genomic prevalence was 5.8 (Supplementary Table 6).

## Discussion

We conducted the first systematic analysis to estimate PLPVs using a global genomic database. One-third of the PLPVs were previously reported in databases and literature, and the remaining two-thirds were manually classified by assessing their pathogenicity according to the 2015 ACMG guidelines. Therefore, we believe that we accurately analyzed the pathogenicity of all variants and the selected PLPVs.

Our study revealed that the allele frequency ratio for the literature verified PLPVs compared to the total PLPVs is high in European populations, including ASJ, NFE, and FIN, compared with the non-European population including AMR, SAS, EAS, and AFR. This finding is consistent with previous observations, indicating the limited knowledge of genetic diversity outside European populations^12,13^.

Our study revealed that the predicted genetic prevalence of AR-NMDs was 24.3 per 100,000 individuals. This result is lower than the prevalence of genetic neuromuscular diseases ranging from 28.6 to 82.8 per 100,000 individuals in previous studies^3,4^. However, considering that these studies included other neuromuscular diseases^3,4^, our results showed that the prevalence of AR-NMDs was significantly higher than expected. This conclusion is based on the following evidence. First, the major common causative genes of genetic neuromuscular diseases are not inherited in an autosomal recessive manner. X-linked dystrophinopathy, autosomal dominant myotonic dystrophy, and autosomal dominant facioscapulohumeral muscular dystrophy account for more than 27% of all patients with genetic myopathy^5^. Additionally, autosomal dominant or X-linked hereditary motor and sensory neuropathy account for 96–98% of patients with genetically-confirmed cases^14,15^. Second, our analysis did not include both deletions or duplications of one or more exons. For example, homozygous absence of exon 7 in *SMN1* is found in approximately 95% of patients with spinal muscular atrophy, the most common disease of genetic motor neuron disease^16^. Third, the diagnostic rates of exome sequencing and genome sequencing are only 30–40% in patients with Mendelian diseases including genetic neuromuscular diseases^1^. Conversely, two-thirds of patients with genetic neuromuscular diseases cannot be identified using exome and genome sequences sourced from the gnomAD database.

Our study revealed that carrier frequency is high in the NFE and AFR populations, but the predicted genetic prevalence is high in the ASJ and EAS populations. This is because the number of genes with carrier frequencies exceeding 1% in the ASJ and EAS populations is four and three, respectively, which is higher than that in other populations.

Our results revealed that the most common causative gene of AR-NMD is *GAA* in the global population. One previous study showed that *SMN1* and *GAA* is the most common causative genes of AR-NMDs in 108 autosomal recessive Mendelian diseases^17^. However, we could not analyze large exonal deletions, the main alterations in *SMN1* in this study. This is likely why *GAA* emerged as the most common AR-NMD gene. This previous study showed that the carrier frequency of individuals with PLPVs in *GAA* was 0.8%, which is slightly lower than our results^17^. This study also indicated that the carrier frequency of PLPVs in *GAA* was much lower in the EAS population (0.3%) than in the European population (0.9%)^17^. This contrasts our results, which found similar frequencies in both populations. This was because the [c.752C>T; c.761C>T] variant, the most common PLPV in the EAS population, was not found or was classified as a variant of uncertain significance in this study. This variant is currently considered the major PLPV in *GAA*, especially in the EAS population, but has been frequently classified as a variant of uncertain significance^18^. Our predicted genetic prevalence was somewhat consistent with the results from newborn screening programs, which reported a prevalence ranging from 3.6 to 11.5 per 100,000 individuals^11,19–22^. Additionally, one previous study used the same analytical method to assess the carrier frequency and genetic prevalence of individuals with PLPVs in *GAA*^11^. The results reported a carrier frequency of 1.3% and genetic prevalence of 4.3 per 100,000 persons in *GAA*, which is nearly identical to our results^11^. The most common PLPVs in *GAA* vary by ethnicity, with the c.-32-13T>G and [c.752C>T; c.761C>T] variants being most common in the NFE and EAS population, respectively. These findings are consistent with previous study^11^.

Our findings on the common causative genes in the EAS, NFE, ASJ, FIN, and SAS populations were consistent with previous results. In the NFE population, the most common causative gene is *GAA* and the most common PLPV is c.-32-13T>G in *GAA*, which is supported by several studies^11,17^. However, our predicted genetic prevalence was higher than previous prevalence data (1.7–2.5 per 100,000) for the United States and Dutch populations^23,24^. In the ASJ population, *FKTN* is well-known to be associated with the common founder variant, c.1167dupA^25^. The carrier frequency of this variant was 0.0160, which is consistent with our results (0.0156)^26^. In the EAS population, *GAA* was the most common AR-NMD gene, which is consistent with previous results in the Chinese population^27^. One study showed the carrier frequency of *GAA* ranged from 0.01 to 0.005^28^. Another recent study showed that the carrier frequency of individuals with PLPVs in *GAA* in Chinese population was 0.0145, which is similar to our result (0.0158) in the EAS population^27^. Lethal congenital contracture syndrome and lethal arthrogryposis with anterior horn cell disease associated with *GLE1* were first reported in FIN population^29^. In particular, c.433-10A>G in *GLE1*, called Fin_Major_, is a representative pathogenic variant in *GLE1* that is commonly observed in the Finnish population^29,30^. Additionally, the prevalence of individuals with PLPVs in *GAA* are relatively low in the FIN population compared with the NFE population^31^. In the Indian population, a representative group of the SAS population, large-scale genetic analysis showed that the most common causative gene of genetic myopathy was *GNE*, which is consistent with our result for the SAS population^32^. Additionally, c.2179 G>A in *GNE* is a founder and major PLPV in the Indian population, which is consistent with results from previous studies^32,33^.

Our findings on the common causative genes in the AFR and AMR populations differ from those of previous studies. However, there have been few large-scale genetic analyses of these populations compared to those in the European population. Our results showed that *PGAM2* is the most common gene in the AFR population. However, no study has investigated the prevalence of individuals with alterations in *PGAM2*. Muscle phosphoglycerate mutase deficiency caused by alterations in *PGAM2* are frequently found in African-Americans^34^. In particular, c.233G>A in *PGAM2* is a founder variant in the African-American population, which is consistent with our result^34^. One epidemiological study in the Moroccan population showed that the carrier frequency of the c.525del pathogenic variant in *SGCG* was 4%, which contrasts our result (1%) in the AFR population^35^. However, because the Moroccan population is a small subset of the AFR population, the results of two studies cannot be directly compared. In the AMR population, our study showed that *ANO5* was the most common causative gene, and the c.692G>T variant in *ANO5* was the second most common PLPV. This variant in *ANO5* is a common PLPV in the European population but is different from the c.191dupA and c.2272C>T variants, which are the most common variants in the Northern European and FIN populations, respectively^36,37^. Several molecular genetic studies have shown that *DYSF* and *CAPN3* are more prevalent as causative genes than *ANO5* in patients with limb-girdle muscular weakness in the Latino, Chilean, or Argentine populations^38–40^. Analysis of common causative genes of AR-NMD in the AFR and AMR populations requires additional large-scale studies.

The number of individuals from the gnomAD database with PLPVs was approximately five times higher than the expected number of individuals with homozygous PLPVs. This finding suggests that the gnomAD database includes patients with AR-NMD and other possible genetic diseases, which is consistent with the results of a previous study^8^. Additionally, this discrepancy might lead to an overestimation of the allele frequency of PLPVs, despite the small number of homozygous gnomAD individuals (28 of 141,456).

Our study had several limitations. First, we identified carriers and individuals by focusing on genes and PLPVs known to cause AR-NMDs. Therefore, our study could not analyze patients with alterations in unidentified causative genes. Furthermore, we posit that many disease-causing variants, particularly non-truncating variants that have not been documented in literature, are classified as variants of unknown significance. Second, we could not analyze large deletions or duplications of exons because of the nature of the gnomAD database. Third, we calculated the predicted genetic prevalence based on the Hardy–Weinberg equation. Therefore, the actual prevalence of AR-NMDs may be higher than the values obtained in the African and South Asian populations, which have high levels of consanguinity and intracommunity marriages^41^. Forth, this study analyzed gnomAD data, next-generation sequencing data that has not been verified through Sanger sequencing.

In summary, our study offers a comprehensive analysis of the carrier frequency and predicted genetic prevalence of AR-NMDs in the global population and six major subpopulations. These results provide crucial insights for the epidemiological analysis, genetic counseling, newborn screening, diagnostic approaches, and therapeutic development of AR-NMDs. We found that the carrier frequency of AR-NMDs was higher than expected, constituting approximately one-third of the entire human population. Furthermore, our findings highlight the heterogeneity of genetic susceptibility to AR-NMDs based on ethnicity.

## Materials and methods

### Selection of AR-NMD genes

Based on the gene table of neuromuscular diseases available at (https://www.musclegenetable.fr), we identified 584 genes associated with neuromuscular diseases, including muscular dystrophies, congenital muscular dystrophies, congenital myopathies, distal myopathies, other myopathies, myotonic syndromes, ion channel muscle diseases, metabolic myopathies, hereditary cardiomyopathies, congenital myasthenic syndromes, motor neuron diseases, hereditary ataxia, hereditary motor and sensory neuropathies, and hereditary paraplegias. We then selected genes linked to AR-NMDs. Among these, *LAMA5* and *LAMB2* were excluded for the following reasons: *LAMA5* is associated with various multisystem syndromes including neuromuscular diseases, epilepsy, and nephropathy; *LAMB2* is associated with congenital myasthenic and nephrotic syndromes. However, although *CAPN3* is associated with autosomal dominant and recessive inheritance patterns, it was included because it is a common causative agent of AR-NMDs. A total of 268 genes linked to AR-NMDs were selected (Supplementary Table 1).

### Analysis of pathogenicity of variants from the gnomAD database

We analyzed genetic variants derived from a dataset of 125,748 exome sequences and 15,708 genome sequences, sourced from the gnomAD database (version 2.1.1; https://gnomad.broadinstitute.org). This dataset comprised individuals from diverse populations: 12,487, 17,720, 5185, 9977, 12,562, 64,603, and 15,308 from the African/African-American (AFR), Latino/Admixed American (AMR), Ashkenazi Jewish (ASJ), East Asian (EAS), Finnish (FIN), non-Finnish European (NFE), South Asian (SAS) populations, as well as 3614 from individuals categorized as “other.”

We organized all the variants associated with AR-NMD genes, sourced from the gnomAD database, into distinct categories (Fig. 1). This classification involved two main subgroups: truncating variants, such as frameshift, splice-site, nonsense, and start-loss variants, and non-truncating variants, including missense, intron, in-frame-deletion/duplication, 5ʹUTR, and 3ʹUTR variants. For further analysis, we initially separated the truncating variants based on their allele frequencies, using a cutoff threshold of 0.005, and divided the non-truncating variants into two subgroups, those with and without references in the scientific literature. Subsequently, we stratified these non-truncating variants using an allele frequency threshold of 0.005.

For literature verified variants, we compiled relevant data from scientific literatures and pertinent databases, such as ClinVar (https://www.ncbi.nlm.nih.gov/clinvar/), HGMD (http://www.hgmd.cf.ac.uk/ac/), and LOVD (https://databases.lovd.nl/shared/genes). Variants lacking representation in scientific literature underwent a comprehensive manual assessment to ascertain their pathogenicity. The process of identifying PLPVs was performed in accordance with the 2015 guidelines of the American College of Medical Genetics and Genomics and the Association for Molecular Pathology^42^. If the total allele counts at the genomic position of the variants were < 1000, they are not classified as PLPVs. This is because variants with very low allele numbers may have a large effect on overall allele frequency.

### Analysis of allele frequency, carrier frequency, and predicted genetic prevalence

The gnomAD database provides specific values for each subpopulation, including allele count, allele number, and homozygote count. Using these values, we calculated the allele and carrier frequencies for a single variant. These calculations were based exclusively on heterozygous PLPVs, as previously described^8^:

$$\begin{aligned} & {\text{Allele frequency}}\;{\text{for a single variant}} = \left( {{\text{allele count}} - 2 \times {\text{homozygous count}}} \right){\text{/allele number}} \\ & {\text{The carrier frequency for a single variant}} = 2 \times \left( {{\text{allele count}} - 2 \times {\text{homozygous count}}} \right){\text{/allele}} \\ & {\text{number}} = 2 \times {\text{allele frequency}} \\ \end{aligned}$$

Subsequently, we calculated the carrier frequency and predicted the genetic prevalence at the genetic level, as previously described^8^:

$$\begin{aligned} & {\text{Carrier frequency at the gene level}} = 1 - \prod\limits_{{{\text{i}} = 1}}^{{\text{n}}} \left( {1 - {\text{carrier frequency for a single variant}}} \right) \\ & {\text{Predicted genetic prevalence at the gene level}} = \sum\limits_{{{\text{k}} = 1}}^{{\text{n}}} \left( {\text{carrier frequency for a single variant}} \right)_{{{\text{ik}}}} \\ \quad \times ({\text{carrier frequency of a single variant}})_{{{\text{ik}}}} \\ \end{aligned}$$

Finally, we determined whether gnomAD included individuals who might have been affected by AR-NMDs but were still asymptomatic, or whose condition was not immediately recognized. To do this, we compared the number of gnomAD individuals with homozygous PLPVs and the expected number of individuals with homozygous variants, calculated using a previously described method^8^.

$$\begin{aligned} & {\text{Expected number of patients with homozygous PLPVs}} = \left\{ \sum\limits_{{{\text{k}} = 1}}^{{\text{n}}} {\left( {\text{carrier frequency for a single variant}} \right)_{{\text{k}}}^{{2}} } \right\} \\ & \quad \times {\text{total individuals}}({141},{456}){\text{ in thegnomAD database version 2}}.{1}.{1}. \\ \end{aligned}$$

### Ethnical consideration

This study was approved by the Institutional Review Board of the Gangnam Severance Hospital, Korea (approval number: 3-2023-0065). The requirement for written informed consent was waived by the board because of all data were provided by the gnomAD with all personal information anonymously encrypted according to a strict confidentiality protocol.

### Supplementary Information

Supplementary Tables.

## Data Availability

Public datasets used in the study are described in the Methods section. All data are available in the manuscript.
